# Mr. Hodgson's Case of Rabies

**Published:** 1808-12

**Authors:** Luke Hodgson

**Affiliations:** No. 9, Giltspur Street, West Smithfield


### *To the Editors of the Medical and Physical Journal.*

GENTLEMEN,

HYDROPHOBIA is at all times an important subject, but more especially when it has been attended to with that diligence, and related with that accuracy which marked the case of Mrs. Chandler, who died in St. Bartholomew's Hospital on Wednesday morning, August the 10th, 1808, and whose case has been given so accurately by Dr. Powell.

I am induced to submit to your consideration, an account of the disease and death of her child, three months old, especially as no event of the kind has, I believe, been recorded.

It is not yet, I think, ascertained whether the human subject can communicate the venom which produces hydrophobia, still less whether such communication can effect a child sucking at the breast of its mother whilst under the disease.

On Monday, August the 8th, eight days subsequent to the removal of the child from the breast, I was requested by the nurse of the child's ward, in the workhouse of St. Sepulchre, London, to see a child who had been seized with convulsions, to which I immediately attended.—On examining the child, there appeared spasmodic affection, especially about the eyes; her stools were black. I ordered medicines, which relieved the bowels; but on the following day, certain appearances about the head induced me to communicate my doubts to Dr. Powell, whether the mother's disease could be the cause of the child's. He did me the honour of seeing her on the Wednesday, and gave it as his opinion, that the child's disease was not hydrophobia.

I saw it on Thursday the 11th with Mr. Tuckwell; it had screamed in the night, there was much excitement, a quick pulse, and heat, but no marked appearance of the disorder in his opinion. The bowels remained better.

Friday, the symptoms increased, and the bowels were again unwell. I had recourse to the same medicines, which relieved her, but in the evening she was seized with (as described by the nurse) staring and fixed eyes, difficulty of swallowing, with a vomiting and a frothy appearance from the mouth.

Saturday, the symptoms about the head remained the same; the bowels were quiet, and the motions nearly natural as to colour and consistency.

Sunday morning, it screamed; the other symptoms much the same.

Monday, the symptoms of the head were much increased, and the whole body put on an appearance which prognosticated dissolution.

Tuesday, when I saw her, strabismus had come on; the eyes, particularly the left, seemed insensible to light, and one side was much warmer than the other.

I requested Mr. Clarke to see it, and took a tea-spoon filled with water and poured it into its mouth. It passed the œsophagus, but we were both of opinion that giving the water produced spasmodic affection.

I saw it in the evening with Mr. Wheeler, who was of opinion, there was no distinct sign of hydrophobia, and at eight o'clock she died.

Ap-

### Appearances on Dissection.

On Thursday, about one o'clock, she was opened; the vessels of her head, like her mother's, were over-charged, there was more water than usual in the ventricles; the whole abdominal and thoracic viscera were perfectly healthy.

It was agreed that hydrophobia was not identified in the child, but it might be said there had been much nervous irritability, and the only doubt is, whether such irritability might not be occasioned by the mother's disease.

In this case, we find a child in perfect health, until she is taken from the mother, who has suffered death from the bite of a mad cat, which death is not produced till thirty days after she is bitten.

The strabismus, a diagnostic sign of hydrocephalus, appears on the ninth day, but the increased actions on the fourth.

It is also to be remarked, that upon dissection, we find no distant cause from the appearance of the bowels, or any other part of the viscera, that hydrocephalus could produce, as they were in a most healthy state.

The difficulty of swallowing on Friday, and the vomiting which followed, together with the observation of Mr. Clark and myself, on giving water to the last day, are circumstances by no means unimportant.

Such, Gentlemen, are all the particulars of this case, which I marked as they occurred, and which if you think with me ought to be recorded, are at your service.

I am, &c.

*No. 9, Giltspur Street, West Smithfield,* LUKE HODGSON.

*August* 27, 1808.

